# Prediction of Metabolic Syndrome for the Survival of Patients With Digestive Tract Cancer: A Meta-Analysis

**DOI:** 10.3389/fonc.2019.00281

**Published:** 2019-04-17

**Authors:** Dan Hu, Meijin Zhang, Hejun Zhang, Yan Xia, Jinxiu Lin, Xiongwei Zheng, Feng Peng, Wenquan Niu

**Affiliations:** ^1^Department of Pathology, Fujian Cancer Hospital & Fujian Medical University Cancer Hospital, Fuzhou, China; ^2^Department of Cardiology, First Affiliated Hospital of Fujian Medical University, Fuzhou, China; ^3^Institute of Clinical Medical Sciences, China-Japan Friendship Hospital, Beijing, China

**Keywords:** digestive tract cancer, metabolic syndrome, meta-analysis, survival, prediction

## Abstract

**Background and Objectives:** Growing evidence indicates that metabolic syndrome confers a differential risk for the development and progression of many types of cancer, especially in the digestive tract system. We here synthesized the results of published cohort studies to test whether baseline metabolic syndrome and its components can predict survival in patients with esophageal, gastric, or colorectal cancer.

**Methods:** Literature retrieval, publication selection and data extraction were performed independently by two authors. Analyses were done using STATA software (version 14.1).

**Results:** A total of 15 publications involving 54,656 patients were meta-analyzed. In overall analyses, the presence of metabolic syndrome was associated with a non-significant 19% increased mortality risk for digestive tract cancer (hazard ratio [HR]: 1.19; 95% confidence interval [CI]: 1.45 to 2.520.95 to 1.49, *P* = 0.130; *I*^2^: 94.8%). In stratified analyses, the association between metabolic syndrome and digestive tract cancer survival was statistically significant in prospective studies (HR: 1.64, 95% CI: 1.18 to 2.28), in studies involving postsurgical patients (HR: 1.42, 95% CI: 1.06 to 1.92), and in studies assessing cancer-specific survival (HR: 1.91, 95% CI: 1.45 to 2.52). Further meta-regression analyses indicated that age and smoking were potential sources of between-study heterogeneity (both *P* < 0.001). The shape of the Begg's funnel plot seemed symmetrical (Begg's test *P* = 0.945 and Egger's test *P* = 0.305).

**Conclusions:** Our findings indicate that metabolic syndrome is associated with an increased risk of postsurgical digestive tract cancer-specific mortality. Continued investigations are needed to uncover the precise molecule mechanism linking metabolic syndrome and digestive tract cancer.

## Introduction

The burden of metabolic syndrome is increasing globally, and the clusters of metabolic risk factors differ regionally (1). Emerging evidence from epidemiologic, clinical and experimental studies indicates that metabolic syndrome confers a differential risk for the development and progression of many types of cancer, especially in the digestive tract system (2–4). For example, metabolic syndrome affects over one in four persons with cancer history, yet less than one in five persons without (5). As demonstrated by the Chinese FIESTA cohort, the presence of metabolic syndrome respectively contributed to a 1.45-, 2.30-, and 2.98-fold increase in postsurgical mortality risk of esophageal squamous cell carcinoma (6), gastric cancer (7), and colorectal cancer (8). Additionally, as tumor is highly energy-demanding tissue, metabolic intermediates are required to foster cancer cell proliferation, survival, and metastasis (9, 10). Based on the above lines of evidence, we here developed a hypothesis that metabolic syndrome may be an important prognostic factor for common digestive tract cancer. However, a literature search has failed to reveal any comprehensive evaluation on this hypothesis. As data on the relation between metabolic syndrome and cancer survival are accumulating lately, we therefore decided to synthesize the results of published cohort studies to test whether metabolic syndrome and its individual components can predict survival outcomes in patients with esophageal, gastric, or colorectal cancer.

## Methods

This meta-analysis was conducted according to the guidelines in the Preferred Reporting Items for Systematic Reviews and Meta-analyses (PRISMA) statement (11). The PRISMA checklist is provided in Supplementary Table 1.

### Medical Literature Retrieval

Public databases including Medline (PubMed) and EMBASE were retrieved for potentially eligible publications as of November 14, 2018. Initial restriction was posed on publications written in the English language and involving human participants. The retrieval process was independently completed by two authors (Dan Hu and Wenquan Niu) using the same subject terms, including (colorectal OR colon OR rectal OR gastric OR stomach OR esophageal OR oesophageal) AND (cancer OR carcinoma OR malignancy OR tumor OR tumor OR neoplasm) AND (metabolic syndrome) AND (survival OR prognosis OR hazard ratio OR surgery OR operation). The reference lists of major retrieved articles and systematic reviews were also checked for potential missing hits.

### Inclusion and Exclusion Criteria

Articles were included if they met the following predefined criteria: (1) either retrospective or prospective cohort design; (2) available prevalence of metabolic syndrome at baseline; (3) restriction to esophageal cancer or gastric cancer or colorectal cancer; (4) available overall survival or cancer-specific survival (either crude or adjusted) effect size estimates of metabolic syndrome expressed as hazard ratio (HR) and 95% confidence interval (95% CI).

Publications were excluded if they were ecological studies, cross-sectional studies, narrative, or quantitative reviews, editorials, case reports or series, meeting abstracts, or studies written in the non-English language.

### Data Collection

From each eligible study, relevant data was collected by two authors (Dan Hu and Wenquan Niu) independently, and was typed into a pre-designed Excel spreadsheet template (Microsoft, Redmond, WA, USA), including the first author's name, publication year, ethnicity, study design, cancer type, percentage of patients receiving surgical treatment for esophageal, gastric or colorectal cancer, follow-up period, sample size, age, gender, cigarette smoking, tumor node metastasis (TNM) stage, definition of metabolic syndrome, percentage of metabolic syndrome, survival outcome, adjustment information, effect size with 95% CI for the prediction of metabolic syndrome, and metabolic components (obesity, hypertension, diabetes mellitus, and dyslipidemia).

During data collection, any discrepancy was resolved by a joint re-evaluation of the original article and, when necessary, adjudicated by a third author (Feng Peng).

### Statistical Analyses

Pooled HR and its 95% CI were generated under the random-effects model when assessing the prediction of metabolic syndrome and metabolic components for overall survival or cancer-specific survival of esophageal, gastric or colorectal cancer.

The inconsistency index (*I*^2^) was calculated to quantify the magnitude of between-study heterogeneity, and its value represents the percentage of observed diversity between studies that is a consequence of heterogeneity other than chance. Significant heterogeneity is recorded if the *I*^2^ is greater than 50% (12), with a higher value representing a higher degree of heterogeneity.

Irrespective of the magnitude of statistical heterogeneity, subgroup analyses and meta-regression analyses were employed to seek possible methodological and clinical causes of heterogeneous estimates.

Cumulative analyses were conducted to examine the impact of the first publication on subsequent publications, and the evolution of the accumulated estimates over time.

The probability of publication bias was inspected by use of both Begg's and filled funnel plots from a visual aspect and Egger's regression asymmetry test from a statistical aspect. Publication bias is significant if the *P*-value of Egger's test is less than 10%. In case of significant publication bias, the number of theoretically missing studies was estimated by use of the Duval and Tweedie nonparametric “trim and fill” method.

The STATA software version 14.1 (StataCorp, College Station, TX, USA) was used to perform calculations and draw plots.

## Results

### Qualified Studies

Using preset subject terms, a total of 147 publications were identified, and only 15 of them satisfied the inclusion and exclusion criteria (6–8, 13–24). The PRISMA flow chart is presented in Supplementary Figure 1. As six publications provided effect-size estimates under both univariate and multivariable models (7, 8, 15, 16, 19, 22), they were treated separately in subgroup analyses under different models, and effect-size estimation from the multivariable models was used in overall analysis. In addition, one publication by Peng et al. (6) partitioned data by gender, which was then treated as two independent studies. Thus, 16 independent studies and 54,656 patients were synthesized in overall analyses, and 22 studies in subgroup analyses by model.

### Study Characteristics

Table 1 presents baseline characteristics of all included studies in this meta-analysis. Colorectal cancer was investigated in nine studies (8, 13, 16–18, 20–23), esophageal squamous cell carcinoma in four studies (6, 19, 24), and gastric cancer in three studies (7, 14, 15). All patients were recorded by 11 studies (6–8, 14, 18, 19, 22–24) to undergo the surgery for digestive tract cancer. There were six prospective cohorts (6–8, 16, 18) and 10 retrospective cohorts (13–15, 17, 19–24). Mean or median follow-up periods ranged from 21.3 months (22) to 72 months (18). Five cohorts reported cancer-specific survival, and 11 cohorts reported overall survival. The sample size of individual studies ranged from 142 (23) to 36,079 (13). The percent of cigarette smoking ranged from 10.93 (8) to 63.42% (19).

Table 1Baseline characteristics of all included studies in this meta-analysis.

**Table:** 

**Authors**	**Year**	**Race**	**Cancer type**	**Surgery**	**Design**	**Follow-up (months)**	**Patients (N)**	**Age (years)**	**Men (N)**	**Smoking**	**TNM stage**	**MetS diagnosis**
Liu B	2018	Chinese	ESCC	100%	Retrospective	39.59	519	62.08	425	48.70%	I-III	CDS
Croft B	2018	Canadian	CRC	100%	Retrospective	65.3	142	68.90	70	13.90%	I-III	NA
Chen Z (Un)	2018	Chinese	CRC	100%	Retrospective	21.3	764	50.74	0	NA	I-III	CDS
Chen Z (Mu)	2018	Chinese	CRC	100%	Retrospective	21.3	764	50.74	0	NA	I-III	CDS
Chen D	2018	Chinese	CRC	100%	Retrospective	40.6	838	50.92	838	61.81%	I-III	CDS
You J	2017	Chinese	CRC	87%	Retrospective	71.2	1,163	65.20	700	26.50%	I-IV	CDS
Peng F (M)	2017	Chinese	ESCC	100%	Prospective	38.2	2,396	56.65	1,822	41.82%	I-III	CDS
Peng F (F)	2017	Chinese	ESCC	100%	Prospective	38.2	2,396	56.65	0	41.82%	I-III	CDS
Hu D (Un)	2017	Chinese	GC	100%	Prospective	31.3	3,012	58.62	2,239	18.46%	I-IV	CDS
Hu D (Mu)	2017	Chinese	GC	100%	Prospective	31.3	3,012	58.62	2,239	18.46%	I-IV	CDS
Wen Y (Un)	2016	Chinese	ESCC	100%	Retrospective	42.9	596	58.00	440	63.42%	I-III	ATPIII
Wen Y (Mu)	2016	Chinese	ESCC	100%	Retrospective	42.9	596	58.00	440	63.42%	I-III	ATPIII
Peng F (Un)	2016	Chinese	CRC	100%	Prospective	58.6	1,318	56.37	758	10.93%	I-IV	CDS
Peng F (Mu)	2016	Chinese	CRC	100%	Prospective	58.6	1,318	56.37	758	10.93%	I-IV	CDS
Cespedes F	2016	Mixed	CRC	100%	Prospective	72	2,446	64.00	1,251	54.05%	I-III	AHA
You J	2015	Chinese	CRC	93%	Retrospective	59.6	1,069	67.00	630	25.35%	I-III	CDS
Ahmadi A (Un)	2015	Iranian	CRC	NA	Prospective	25	1,127	54.00	690	NA	I-IV	NA
Ahmadi A (Mu)	2015	Iranian	CRC	NA	Prospective	25	1,127	54.00	690	NA	I-IV	NA
Wei X (Un)	2014	Chinese	GC	93%	Retrospective	NA	587	53.50	406	NA	I-IV	ATPIII
Wei X (Mu)	2014	Chinese	GC	93%	Retrospective	NA	587	53.50	406	NA	I-IV	ATPIII
Kim E	2014	Korean	GC	100%	Retrospective	53.2	204	60.40	143	NA	I-IV	ATPIII
Yang Y	2013	Chinese	CRC	84%	Retrospective	72	36,079	77.90	14,820	NA	I-IV	ATPIII

**Table:** 

**Authors**	**Survival**	**Model**	**Metabolic syndrome**			**Obesity**			**Hypertension**			**Diabetes mellitus**			**Dyslipidemia**		
			**ES**	**LL**	**HL**	**ES**	**LL**	**HL**	**ES**	**LL**	**HL**	**ES**	**LL**	**HL**	**ES**	**LL**	**HL**
Liu B	Overall	Univariate	0.687	0.463	1.018	0.328	0.225	0.478	0.845	0.643	1.111	0.579	0.420	0.799	0.713	0.523	0.971
Croft B	Overall	Multivariate	1.090	0.270	4.300	NA	NA	NA	3.100	0.830	12.220	1.320	0.510	3.400	1.860	0.580	5.970
Chen Z (Un)	Cancer-specific	Univariate	1.621	1.221	2.133	NA	NA	NA	NA	NA	NA	NA	NA	NA	NA	NA	NA
Chen Z (Mu)	Cancer-specific	Multivariate	1.558	1.153	2.012	NA	NA	NA	NA	NA	NA	NA	NA	NA	NA	NA	NA
Chen D	Overall	Univariate	1.210	0.720	1.530	NA	NA	NA	NA	NA	NA	NA	NA	NA	NA	NA	NA
You J	Overall	Univariate	0.932	0.830	1.047	NA	NA	NA	0.860	0.697	1.060	0.997	0.847	1.174	NA	NA	NA
Peng F (M)	Cancer-specific	Multivariate	1.450	1.140	1.830	0.900	0.720	1.120	1.170	0.970	1.400	1.980	1.680	2.330	1.410	1.200	1.650
Peng F (F)	Cancer-specific	Multivariate	1.460	0.920	2.310	1.040	0.710	1.520	0.900	0.600	1.340	1.760	1.230	2.510	1.190	0.840	1.690
Hu D (Un)	Cancer-specific	Univariate	2.530	2.240	2.850	1.330	1.170	1.500	1.210	1.080	1.350	3.360	3.000	3.760	1.850	1.650	2.090
Hu D (Mu)	Cancer-specific	Multivariate	2.300	2.020	2.620	1.280	1.120	1.460	1.400	1.240	1.580	3.260	2.900	3.680	1.960	1.730	2.230
Wen Y (Un)	Overall	Univariate	0.576	0.389	0.854	0.829	0.614	1.119	0.867	0.695	1.082	1.045	0.752	1.453	NA	NA	NA
Wen Y (Mu)	Overall	Multivariate	0.590	0.397	0.877	NA	NA	NA	NA	NA	NA	NA	NA	NA	NA	NA	NA
Peng F (Un)	Cancer-specific	Univariate	3.050	2.480	3.760	1.290	1.050	1.600	1.310	1.080	1.600	4.960	4.060	6.070	2.040	1.669	2.510
Peng F (Mu)	Cancer-specific	Multivariate	2.980	2.400	3.690	1.000	0.990	1.010	1.640	1.320	2.040	5.130	4.180	6.290	2.060	1.670	2.540
Cespedes F	Overall	Multivariate	1.230	1.030	1.560	NA	NA	NA	0.840	0.680	1.030	1.070	0.900	1.260	NA	NA	NA
You J	Overall	Univariate	0.790	0.588	1.062	0.887	0.660	1.191	0.903	0.685	1.191	0.965	0.628	1.483	NA	NA	NA
Ahmadi A (Un)	Overall	Univariate	0.810	0.060	3.780	0.710	0.350	6.800	0.820	0.500	1.640	0.900	0.500	1.640	NA	NA	NA
Ahmadi A (Mu)	Overall	Multivariate	0.950	0.520	1.220	0.620	0.440	8.700	0.830	0.420	1.640	1.450	0.710	2.950	NA	NA	NA
Wei X (Un)	Overall	Univariate	0.584	0.382	0.893	0.893	0.636	1.253	0.862	0.660	1.125	0.888	0.581	1.357	NA	NA	NA
Wei X (Mu)	Overall	Multivariate	0.565	0.368	0.868	NA	NA	NA	NA	NA	NA	NA	NA	NA	NA	NA	NA
Kim E	Overall	Multivariate	2.880	1.340	6.220	NA	NA	NA	NA	NA	NA	NA	NA	NA	NA	NA	NA
Yang Y	Overall	Multivariate	0.980	0.930	1.020	0.990	0.910	1.070	1.080	1.030	1.120	1.170	1.130	1.210	0.770	0.750	0.800

*U, univariate model; Mu, multivariable model; M, male; F, female; ES, effect size; LL, low 95% limit; HL, high 95% limit; CRC, colorectal cancer; ESCC, esophageal squamous cell carcinoma; GC, gastric cancer; TNM, tumor node metastasis; MetS, metabolic syndrome; CDS, Chinese Diabetes Society; AHA, American Heart Association; ATPIII, Adult Treatment Panel III; NA, not available*.

### Overall and Model-Dependent Analyses

**Figure 1:**
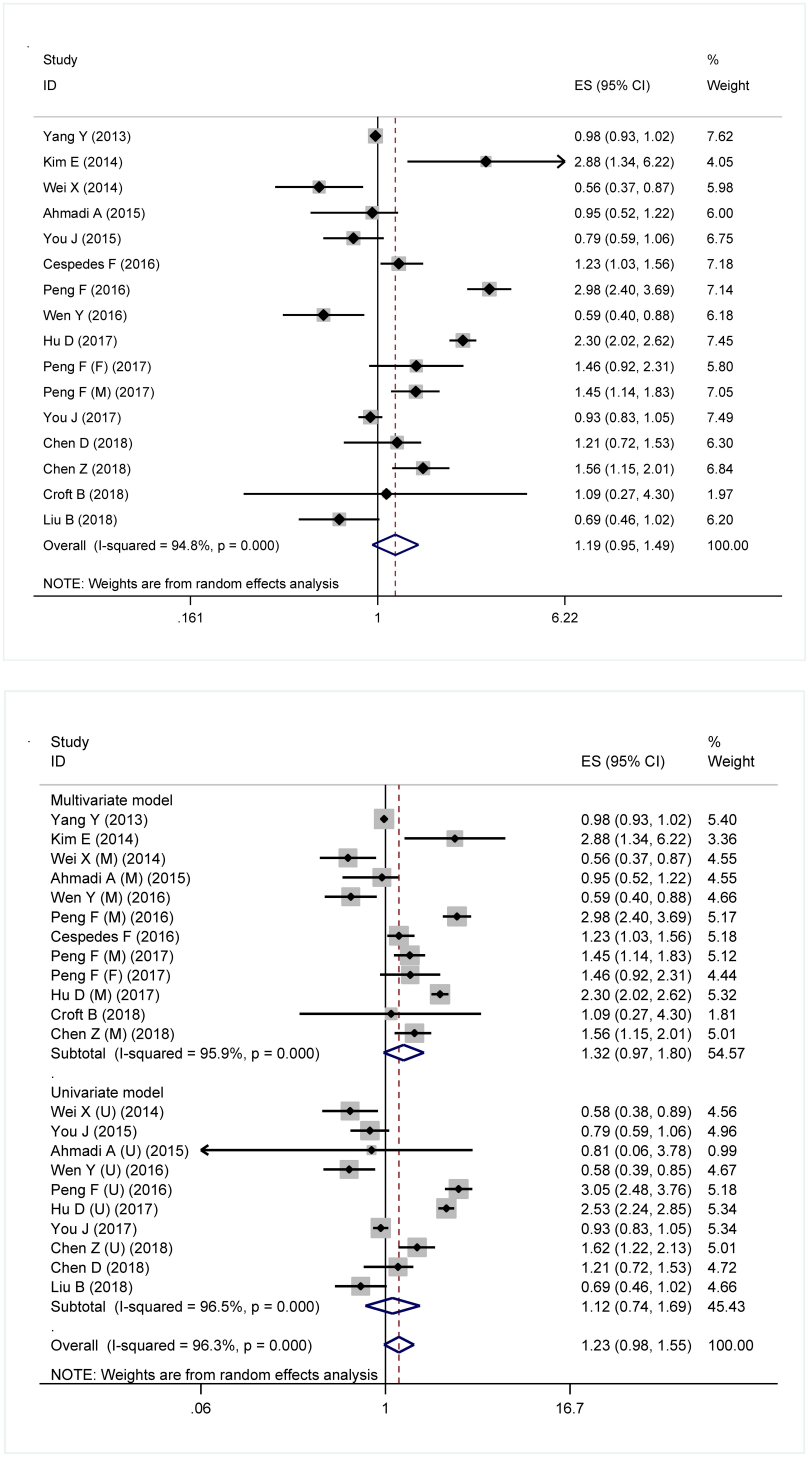
Forest plots for the prediction of metabolic syndrome for the survival of digestive tract cancer overall **(Upper)** and under different models **(Lower)**. ES, effect size; 95% CI, 95% confidence interval.

Figure 1 shows forest plots for the prediction of metabolic syndrome for overall survival and model-specific survival of patients with digestive tract cancer. In overall analysis, the presence of metabolic syndrome was associated with a non-significant 19% increased mortality risk in digestive tract cancer (HR: 1.19; 95% CI: 0.95 to 1.49, *P* = 0.130), and this association was obsessed by significant between-study heterogeneity (*I*^2^: 94.8%) (Figure 1: the upper panel). In model-dependent analysis, effect size estimate was potentiated under the multivariable model (HR: 1.32, 95% CI: 0.97 to 1.80), relative to the univariate model (HR: 1.12, 95% CI: 0.74 to 1.69), and heterogeneity was an obsessing issue for both models (Figure 1: the lower panel).

In addition, effect size estimates of four metabolic components in predicting the survival of digestive tract cancer are summarized in Table 2. Only diabetes mellitus was a significant risk factor for the mortality of digestive tract cancer (HR: 1.51, 95% CI: 1.06 to 2.14), with significant heterogeneity between studies (*I*^2^: 98.0%). Obesity tended to be associated with a reduced mortality risk (HR: 0.93, 95% CI: 0.81 to 1.06).

**Table 2:** Risk estimates of four metabolic components for the survival of digestive tract cancer.

**Metabolic components**	**Studies**	**ES**	**95% CI**	***P***	***I*^**2**^**	***P***
Obesity	8	0.93	0.81 to 1.06	0.276	85.7%	< 0.001
Hypertension	11	1.07	0.93 to 1.22	0.342	80.2%	< 0.001
Diabetes mellitus	11	1.51	1.06 to 2.14	0.023	98.0%	< 0.001
Dyslipidemia	7	1.29	0.83 to 1.99	0.256	98.1%	< 0.001

*ES, effect size; 95% CI, 95% confidence interval; I^2^, inconsistency index*.

### Cumulative Analyses

Cumulative analyses on the prediction of metabolic syndrome for digestive tract cancer survival are shown in Supplementary Figure 2. There is no evidence that the significant findings of the first published study triggered subsequent replications.

### Subgroup Analyses

As significant heterogeneity was observed in overall analyses, a panel of stratified analyses were conducted according to cancer type, complete surgery, total sample size, ethnicity, study design, TNM stage, follow-up period, and survival outcome, respectively (Table 3). By cancer type, there was no detectable significance in effect size for three types of cancer under study, and the risk tendency seemed more obvious for gastric cancer and colorectal cancer. By complete surgery, the presence of metabolic syndrome was significantly associated with a 1.42-fold (95% CI: 1.06 to 1.92) increased mortality risk for patients who underwent the surgery for digestive tract cancer. By sample size, effect size was marginally significant in studies involving over 1000 patients (HR: 1.33, 95% CI: 0.99 to 1.87).

**Table 3:** Stratified risk estimates of metabolic syndrome for the survival of digestive tract cancer.

**Metabolic syndrome**	**Studies**	**ES**	**95% CI**	**P**	***I*^**2**^**	**P**
**Cancer type**						
CRC	9	1.22	0.96 to 1.55	0.112	93.1%	< 0.001
ESCC	4	0.97	0.60 to 1.57	0.897	86.1%	< 0.001
GC	3	1.53	0.95 to 1.49	0.411	94.8%	< 0.001
**Complete surgery**						
No	5	0.91	0.81 to 1.02	0.097	53.0%	0.074
Yes	11	1.42	1.06 to 1.92	0.020	91.0%	< 0.001
**Total sample size**						
< 1000	7	1.00	0.66 to 1.51	0.998	82.9%	< 0.001
≥1000	9	1.33	0.99 to 1.78	0.056	96.8%	< 0.001
**Ethnicity**						
Chinese	12	1.16	0.89 to 1.50	0.282	96.1%	< 0.001
Non-chinese	4	1.29	0.89 to 1.87	0.174	51.3%	< 0.001
**Study design**						
Prospective	6	1.64	1.18 to 2.28	0.003	91.4%	< 0.001
Retrospective	10	0.94	0.84 to 1.11	0.469	76.2%	< 0.001
**Tnm stage**						
I-III	9	1.07	0.85 to 1.36	0.550	76.1%	< 0.001
I-IV	7	1.36	0.93 to 1.99	0.116	97.6%	< 0.001
**Follow-up period**						
< 43 months	8	1.19	0.84 to 1.69	0.326	91.3%	< 0.001
≥ 43 months	8	1.18	0.86 to 1.56	0.262	94.2%	< 0.001
**Survival outcome**						
Cancer-specific survival	5	1.91	1.45 to 2.52	< 0.001	85.8%	< 0.001
Overall survival	11	0.93	0.81 to 1.06	0.270	68.4%	< 0.001

*ES, effect size; 95% CI, 95% confidence interval; I^2^, inconsistency index; CRC, colorectal cancer; ESCC, esophageal squamous cell carcinoma; GC, gastric cancer; TNM, tumor node metastasis*.

Because most studies were conducted in Chinese, all studies were split into Chinese and non-Chinese by ethnicity, and no significance was detected. By study design, the association between metabolic syndrome and digestive tract cancer was statistically significant in prospective studies (HR: 1.64, 95% CI: 1.18 to 2.28). By TNM stage, the risk magnitude was stronger in studies involving patients with stage I-IV than patients with stage I-III. Grouping studies according to the median (43 months) of follow-up periods in all studies, risk magnitude did not differ between the two groups. By survival outcome, significance was found in studies investigating cancer-specific survival (HR: 1.91, 95% CI: 1.45 to 2.52).

### Publication Bias

**Figure 2:**
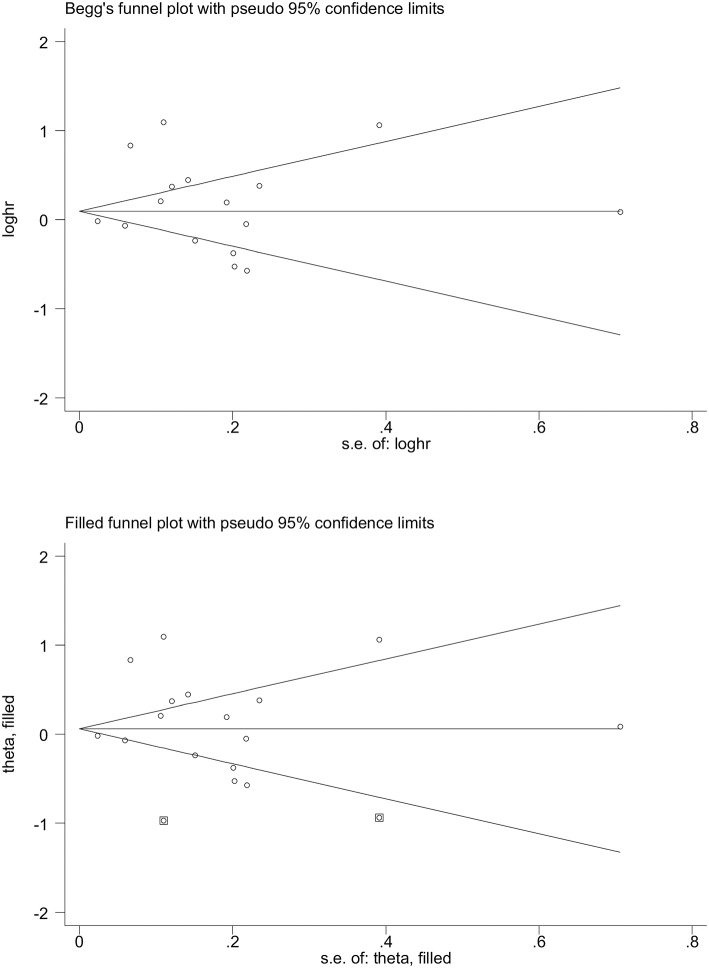
Begg's **(Upper)** and filled **(Lower)** funnel plots for the prediction of metabolic syndrome for the survival of digestive tract cancer. loghr, logarithm of hazard ratio; s. e.: standard error.

Shown in Figure 2 are Begg's and filled funnel plots using all studies to appraise the likelihood of potential publication bias. The shape of the Begg's funnel plot seemed symmetrical (Begg's test *P* = 0.945 and Egger's test *P* = 0.305). As reflected by the filled funnel plot, an estimated two studies were missing to ensure symmetry.

### Meta-Regression Analyses

**Figure 3:**
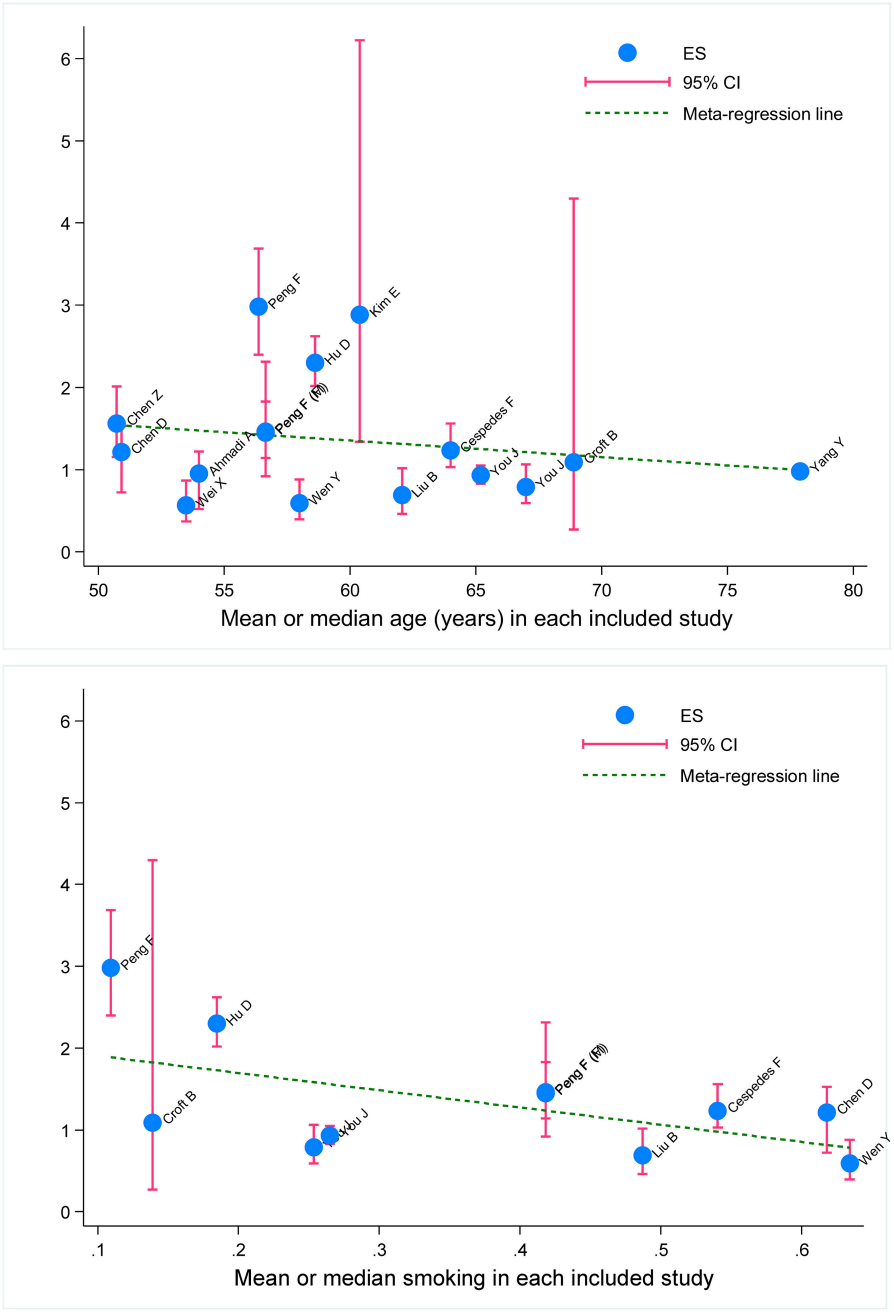
Meta-regression of baseline age and smoking on effect size of metabolic syndrome in prediction of survival of digestive tract cancer. ES, effect size; 95% CI, 95% confidence interval. The vertical coordinate denotes effect size. The blue solid dot represents effect-size estimate, and the vertical pink line represents 95% confidence interval of study. The dotted blue line represents fitted regression line for effect-size estimates.

To explore other possible sources of heterogeneity, meta-regression analyses were conducted on age, gender, and smoking at baseline. Age and smoking were found to be significant confounders (both *P* < 0.001) on the association between metabolic syndrome and digestive tract cancer in all studies (Figure 3).

## Discussion

Via a comprehensive analysis on 15 publications and 54,656 patients, our findings indicate that the presence of metabolic syndrome was associated with an increased risk of digestive tract cancer mortality in overall analyses, and this association was reinforced under the multivariable model, highlighting the independent role of metabolic reprogramming in carcinogenesis. Further stratified analyses indicated statistical significance in studies with a prospective design, involving postsurgical patients or assessing cancer-specific survival outcome. Additionally, as revealed by our meta-regression analyses, age, and cigarette smoking were identified as potential sources of between-study heterogeneity. To the best of our knowledge, this is the first meta-analysis that has comprehensively evaluated the prediction of baseline metabolic syndrome for the survival of three common types of digestive tract cancer.

Metabolic syndrome is increasingly acknowledged as a risk factor for the development and progression of some types of common cancer, such as breast cancer, colorectal cancer, and gastric cancer (4, 23, 25). Although relatively little is known about the exact carcinogenic mechanisms of metabolic syndrome, changes in the expression of transcription and growth factors in the peripheral tissues, as well as in cancer tissues of patients with metabolic syndrome, and changes of bioavailable concentrations of insulin-like growth factor-1 conferred by the influence of hyperinsulinemia, might constitute possible mechanisms (26, 27). By definition, metabolic syndrome is composed of a collection of cardio-metabolic risk factors, including obesity, hypertension, hyperglycemia, and lipid abnormalities (28). In this present study, no statistical significance was observed for metabolic syndrome as a whole, yet its component—diabetes mellitus—was a remarkable risk predictor for poor survival of digestive tract cancer. There is evidence that high glucose may exert direct and indirect effects upon cancer cells to promote proliferation, possibly through increasing the bioactivity of insulin-like growth factor 1 (29). It is also worth noting that the association between metabolic syndrome and digestive tract cancer was statistically significant in prospective studies, in studies involving postsurgical patients, or in studies focusing on cancer-specific survival, indicating that metabolic syndrome might trigger a cascade of carcinogenic reactions that eventually lead to poor survival outcomes. The clinical implications of these findings are noteworthy, particularly the fact that irrespective of the mechanisms, metabolic syndrome, especially diabetes mellitus, can clearly identify and refine digestive tract cancer patients with higher postsurgical risk who could benefit from closer monitoring.

It is widely accepted that exploring possible sources of between-study heterogeneity is a core component of a meta-analysis. Besides study design, surgical treatment, and survival outcome, our further meta-regression analyses indicated that age and cigarette smoking may confound the association between metabolic syndrome and digestive tract cancer survival. As indicated by a clinical study, metabolic syndrome was demonstrated to be a significant and independent predictor for improved survival in patients with old age (15). In a Chinese male cohort, the interaction between smoking and metabolic syndrome can increase the recurrence risk of colorectal cancer (21). Additionally, smoking and metabolic syndrome can significantly impact the prevalence of colorectal cancer, and the diagnostic yields of screening tests in men aged 40 to 49 years. The possible interaction between smoking and metabolic syndrome, however, cannot be investigated here, because individual participant data were not available for this meta-analysis. Moreover, it is important to bear in mind that meta-regression analyses, albeit enabling covariates to be considered, do not have the methodological rigor of a properly designed study that is intended to test the effect of these covariates formally (30).

Several limitations should be acknowledged when interpreting the results, in addition to those inherited from the meta-analysis. Firstly, only published studies written in the English language were retrieved, which might introduce a potential selection bias. Secondly, despite 15 publications involved, in some stratified analyses a small sample size limited the statistical power. Thirdly, differences in the definition of metabolic syndrome and its component might introduce report bias. Fourthly, confounding factors under the multivariable models are not identical, which might bias effect size estimates. Fifthly, as with all meta-analyses, publication bias cannot be ruled out entirely as our analysis is based on publications from the English journals, and the filled funnel plot suggested that a small proportion of small and negative studies were missing.

Taken together, our findings indicate that metabolic syndrome is associated with an increased risk of postsurgical digestive tract cancer-specific mortality. This meta-analysis has the potential to enhance conversations about prognosis and decision making prior to going to surgery. For practical reasons, we hope that this meta-analysis will not remain as just another end point of research, but instead as a beginning to trigger more solid data to understanding the roles of metabolic syndrome and its components in predicting the survival of patients with digestive tract cancer. Additionally, it is necessary for continued investigations to uncover the precise molecule mechanism linking metabolic syndrome and digestive tract cancer because of the major clinical implications.

## Author Contributions

All authors read and approved the final manuscript prior to submission. FP and WN: conceived and designed the experiments; DH, MZ, and WN: performed the experiments; FP and WN: analyzed the data; DH, MZ, HZ, YX, JL, XZ, and WN: contributed materials/analysis tools; FP and WN: wrote the paper.

### Conflict of Interest Statement

The authors declare that the research was conducted in the absence of any commercial or financial relationships that could be construed as a potential conflict of interest.
